# BMP2-Functionalized Biomimetic Calcium Phosphate Graft Promotes Alveolar Defect Healing During Orthodontic Tooth Movement in Beagle Dogs

**DOI:** 10.3389/fbioe.2020.00517

**Published:** 2020-05-29

**Authors:** Shijie Jiang, Tie Liu, Gang Wu, Wen Li, Xiaoxia Feng, Janak L. Pathak, Jiejun Shi

**Affiliations:** ^1^Department of Orthodontics, The Affiliated Stomatology Hospital, Zhejiang University School of Medicine, Hangzhou, China; ^2^Key Laboratory of Oral Biomedical Research of Zhejiang Province, Zhejiang University School of Stomatology, Hangzhou, China; ^3^Department of Oral Implantology, The Affiliated Stomatology Hospital, Zhejiang University School of Medicine, Hangzhou, China; ^4^Department of Oral Implantology and Prosthetic Dentistry, Academic Centre of Dentistry Amsterdam (ACTA), Vrije Universiteit Amsterdam and University of Amsterdam, Amsterdam, Netherlands; ^5^Key Laboratory of Oral Medicine, Guangzhou Institute of Oral Disease, Affiliated Stomatology Hospital of Guangzhou Medical University, Guangzhou, China

**Keywords:** biomimetic calcium phosphate granules, deproteinized bovine bone (DBB) graft, orthodontic tooth movement, alveolar defects, bone regeneration

## Abstract

**Background:** Grafting of biomaterial in alveolar defect facilitates bone healing and orthodontic treatment. BMP2-functionalized biomimetic calcium phosphate (BioCaP) graft had shown excellent bone defect healing potential in many preclinical studies. In this study, we aimed to investigate the influence of BioCaP graft on surgical alveolar bone defect healing during orthodontic tooth movement (OTM) in beagle dogs.

**Methods:** Nine Beagle dogs were randomly assigned to three groups: control, deproteinized bovine bone (DBB), and BioCaP. The maxillary second premolars were protracted into the defects of the extracted maxillary first premolar for 8 weeks. The rate of OTM, alveolar remodeling and bone defect healing were evaluated by histology, histomorphometry, and cone beam computed tomography (CBCT) imaging. Periodontal probing depth was analyzed. Gingival cervicular fluid was collected at week 4 and 8, and the IL-1β level was measured by ELISA.

**Results:** The histological sections of the bone defect showed more newly formed bone in the BioCaP group. The percentage of new bone formation in the BioCaP group was 1.61-, and 1.25-fold higher compared to the control and DBB group, respectively. After 8 weeks of OTM, the resorption rate of BioCaP was 1.42-fold higher compared to DBB. The root resorption index in the DBB group was 1.87-, and 1.39-fold higher compared to the control and BioCaP group, respectively. CBCT images showed 1.92-, and 1.36-fold higher bone mineral density in the BioCaP group compared to the control and DBB group, respectively. There was no significant difference in OTM among the three groups. The distance between the enamel cementum and the crest of the alveolar ridge in the control group was 1.45-, and 1.69-fold higher compared to DBB and BioCaP group, respectively. Periodontal probing depth at week 8 was reduced in the BioCaP group compared to the control. IL-1β concentration in the gingival cervicular fluid was significantly lower in the BioCaP group compared to the control group at week 4 and 8.

**Conclusion:** BioCaP graft robustly promoted bone regeneration and alveolar bone defect healing without affecting OTM. BioCaP graft caused less alveolar bone recession and root resorption of traction tooth with favorable periodontal attachment level indicating that BioCaP as a bioactive and functional bone filling material for alveolar bone defects during orthodontic treatment.

## Introduction

Alveolar bone defects are frequently encountered during orthodontic treatment. The alveolar defects during orthodontic treatment are mainly caused by the removal of neighboring teeth, surgical-orthodontic treatment, and severe periodontitis. Grafting of biomaterials in alveolar defects before orthodontic tooth movement (OTM) had shown successful bone regeneration during orthodontic treatment (Hossain et al., 1996). Previous studies had confirmed that the bone-filling materials facilitate teeth movement and stimulate alveolar bone deposition under orthodontic force (Hossain et al., 1996; Yilmaz et al., 2000; Oltramari et al., 2007; Klein et al., 2019; Nagy et al., 2019). Therefore, the cost-effective alveolar bone defect-filling materials are in high demand in orthodontic clinics.

Alveolar bone surgery can speed up orthodontic tooth movement (Hibino and Wong, 2009; Kim et al., 2015b). Usually, the orthodontic force is applied for at least 3 months after alveolar bone healing (Mayer et al., 1994; Nakamoto et al., 2002). Teeth traction force can be applied immediately to accelerate OTM after the graft material is placed into the surgical defect (Ahn et al., 2014). The early stage of OTM after material grafted in alveolar surgical defect promotes bone regeneration (Ahn et al., 2014). Bone graft materials and techniques accelerate tooth movement into the alveolar defect with healthy bone regeneration (Reichert et al., 2010; Ahn et al., 2014; Tsai et al., 2017). In the case of bone grafting during orthodontic treatment, bone regeneration, graft stability, and any side effects around orthodontic teeth or the periodontium should be meticulously evaluated (Hossain et al., 1989, 1996; Sheats et al., 1991; Araujo et al., 2001; Kawamoto et al., 2002). The satisfactory outcome of early OTM following regenerative surgery suggests that the biomechanical stimulation may not jeopardize the regenerative effect (Tsai et al., 2017). Therefore, the ideal bone grafting materials for orthodontic treatment should have the potential to protect the orthodontic teeth and enhance alveolar bone regeneration.

BMP2 is a potent osteogenic growth factor that also promotes alveolar bone regeneration (King et al., 1997). Literature had reported various BMP2-loaded biomaterials for alveolar bone regeneration (Selvig et al., 2002; Rao et al., 2013; Oortgiesen et al., 2014). However, the clinical use of BMP2-loaded biomaterials for alveolar bone regeneration is still controversial mainly due to the adverse effects of burst released high dose BMP2 (James et al., 2016). We have developed BMP2-functionalized biomimetic calcium phosphate (BioCaP) granules with excellent biocompatibility, osteoconductivity, and osteoinductivity (Liu et al., 2014, 2017; Wang et al., 2019). Biomimetic coating of BMP2 and calcium phosphate onto the surface of the material is an attractive approach for controlled release of BMP2 (Wernike et al., 2010; Wang et al., 2017). BioCaP has shown sustained release of BMP2 both *in vitro* and in bone defect site (Zheng et al., 2014; Liu et al., 2017, 2018). Moreover, BioCaP promoted critical size bone defect healing in both small animal and large animal models (rats, rabbit, dog, and sheep) in different anatomical sites (cranial defects and femoral defects) (Liu et al., 2010, 2014, 2017, 2018; Wang et al., 2017, 2019). One cubic centimeter (1 mL) of BioCaP granules loads 140 μg of BMP2 and robustly promotes critical size bone defect healing (Liu et al., 2017, 2018; Wang et al., 2017). A dose of 1.5 mg/ml of rhBMP2 was identified as the most effective concentration for *de novo* Bone (Boyne et al., 1997, 2005). BioCaP bone graft reduces the dose of BMP2 required for *in vivo* bone regeneration by 10-fold (Boyne et al., 1997, 2005; Liu et al., 2017, 2018; Wang et al., 2017). That could minimize the adverse effects of high dose BMP2 during clinical uses as well as reduced the economic burden of expensive rhBMP2. Therefore, the BioCaP granules could be a promising bone graft for alveolar bone defect healing during orthodontic treatment. However, the potential of BioCaP to heal alveolar bone defect during orthodontic treatment and its effect on OTM has not been investigated yet.

The aim of this study was to analyze the efficacy of BioCaP-graft on surgical alveolar bone defect healing during orthodontics tooth movement (OTM) in a large animal model. We analyzed surgical alveolar bone defect healing in the canine orthodontic treatment model. And DBB bone graft was used as a standard control. We studied the possible effect of BioCaP bone graft on OTM. BioCaP granules robustly enhanced alveolar bone regeneration and preserved the root resorption of OTM teeth compared to DBB. Moreover, the BioCaP bone graft in alveolar defect adjacent to OTM teeth did not obstruct the OTM, reduced inflammation, and preserved periodontal tissue. Our findings indicate BioCaP as a promising osteoinductive biomaterial to improve alveolar bone regeneration during orthodontic treatment.

## Materials and Methods

### Fabrication of BioCaP

BioCaP was fabricated by refining a well-established biomimetic mineralization technique described in our previous studies (Wu et al., 2011; Liu et al., 2013, 2014, 2017). Briefly, a CaP solution (200 mM HCl, 20 mM CaCl_2_·2H_2_O, 680 mM NaCl, and 10 mM Na_2_HPO_4_) was buffered by TRIS (250 mM) to a pH of 7.4. In order to sterilize the CaP solution, it was filtered with a vacuum filter (0.22 μm pore) before buffering. All the following procedures were performed under aseptic conditions. After buffering, the solution was incubated in a shaking water bath (50 agitations/min) at 37°C for 24 h. The solution was removed, and the precipitated material was gently washed by Milli-Q water, filtered, and compressed to a block using a vacuum exhaust filtering (0.22 μm pore, Corning, NY, USA). After drying at room temperature for 2 h, the compacted block was ground and filtered in metallic filter mesh to obtain different sizes (250–1,000 μm) of granules.

### Biomimetic Coating and BMP2 Incorporation

The superficial coating of calcium and phosphate was deposited on BioCaP according to the procedure described before (Liu et al., 2005; Wu et al., 2010). Briefly, the coating solution, 40 mM HCl, 4 mM CaCl_2_·2H_2_O, 136 mM NaCl, 2 mM Na_2_HPO_4_, and 50 mM TRIS (pH 7.4) in total volume of 20 ml was prepared. BioCaP was incubated in the coating solution in a shaking water bath (50 agitations/min) at 37°C for 24 h. The protein BMP-2 (INFUSE® Bone Graft, Medtronic, USA) was added in the supersaturated solution (coating solution) of calcium phosphate at a final concentration of 1 μg/ml, and was subsequently co-precipitated into the biomimetic calcium phosphate coating of the BioCaP granules. The samples were then freeze-dried and characterized as described previously (Zheng et al., 2014; Liu et al., 2017). The entire procedure was conducted under sterile conditions. BioCaP granules with a diameter of 250−1,000 μm were selected in this experiment, which is the size of DBB granules used in this study.

### Experimental Groups and Animal Model

All *in vivo* experiments of this study were carried out in accordance with the principles of the Basel Declaration and recommendations of Zhejiang University Laboratory Animal Center. The protocol was approved by the Ethics committee of the Zhejiang University Laboratory Animal Center (ethic approval number: ZJU20190057, approval date: 2019.05.20). Nine male Beagle dogs (1 year old, 11–13 Kg body weight) were housed in separate cages in the SPF level large animal room of Zhejiang University Laboratory Animal Center. Total 18 examples of maxillary second premolars were randomly allocated to three equal groups to receive the following treatments: Control group (no treatment-negative control), BioCaP group (experiment group), and DBB group (positive control group).

After intraperitoneal anesthesia with 3% pentobarbital sodium (1 ml/kg body weight), dental models of all groups were taken from alginate impression materials (Harbin Dental Equipment Factory, China) and sent to the processing plant (Technical Center of Dental Hospital Affiliated to Medical College of Zhejiang University to produce orthodontic traction devices. Before surgery, the alveolar region of each dog was scanned for CBCT images using NEWTOM 3G QR-DVT9000 CBCT machine (QR r.s.l, Verona, Italy).

Under anesthesia, each dog was fixed on the experimental table in the supine position and injected with the acute infiltration anesthesia Primacaine (SATELEC, France) into the gingival of first and second premolar on both sides of the maxillary. After skin disinfection (0.5% iodophor solution) to the operation sites, the gingival of bilateral maxillary first premolars were cut, and the gingival flaps were pushed to the buccal side. After extraction of the first premolars, a 4.5 mm diameter, and 6 mm deep bone defect was prepared by using a slow-speed dental motor (Korea, MARATHON-3) crack drill at the extraction wound. This defect did not contact with the root of maxillary second premolar. About 110 mm^3^ volume of BioCaP and DBB granules were implanted in the Bio-CaP and DBB group, respectively. The control group defect was left empty. The implant was covered with Bio-Guide (Geistlieh AG, Switzerland) and sutured the gingival flap at the bone defect. Penicillin (500 U/day) was injected intramuscularly up to 3 days after surgery.

### Orthodontic Traction

CBCT image was taken after 1 week of surgery. Orthodontics appliance was bonded on the maxillary arch, and the distance from the fourth premolar cervix to the second premolar cervix of each side was measured by vernier caliper. The orthodontic appliance consisted of a 0.019 × 0.025 inch stainless steel square wire as sectional archwire with tip back for bodily movement of the second premolar, attached with standard tubes (0.022 × 0.028 inch) on the maxillary canine and second premolar. The maxillary second premolar was pulled by a 0.012 inch nickel-titanium closed coiled spring (GRIKIN Advanced Material Co., Ltd., China) to the alveolar bone defect (mesial direction) using the canine teeth as an anchorage. The nickel-titanium closed coiled spring was pulled to until the Orthodontic dynamometer force value is 150 g, and was fixed on the orthodontic appliance by a 0.010 inch ligation wire. After traction, soft food was served. The orthodontic appliances were checked every 3 days to make sure they were not debonding. The distance from the cervix of maxillary 4th premolar to the cervix of maxillary 2nd premolar of each dog was measured every 2 weeks. The traction force was added to maintain the pulling force generated by the coil spring. The change of the distance from the maxillary fourth premolar cervix to maxillary second premolar cervix was considered as the OTM (Kim et al., 2015a). Periodontal probing depth analysis of orthodontic tooth indicates the status of periodontal tissue (Hung and Douglass, 2002). Periodontal probing depth of the maxillary second premolar was measured at the mesial side (mesial-buccal side, mid-buccal side, distal-buccal side, mesial-palatal side, mid-palatal side, and distal-palatal side) in every 2 weeks.

### Level of IL-1β in Gingival Crevicular Fluid

Gingival crevicular fluid (GCF) is a biological exudate from periodontal tissue and its concentration of pro-inflammatory cytokines, including interleukin-1β (IL-1β) indicates alveolar bone resorption, and inflammation (de Aguiar and Perinetti, 2017). After 4 and 8 weeks of OTM, GCF was collected from the mesial gingival crevice of the second premolar using a no. 30 sterile paper point (Javed et al., 2014). The volume of GCF and the concentration of IL-1β were analyzed. Briefly, the sterile paper point was inserted in the mid-buccal gingival crevice of the orthodontic tooth and held in place for 60 seconds. The GCF volume density was considered as 1, and the volume of GCF was showed by the difference in weight. After sample collection, paper points were transferred to sterile microcentrifuge tubes and stored in −80°C refrigerator. The adsorbed volume of GCF in each paper point was weighed using an analytic digital balance before and after collection immediately. Each paper point was eluted by 80 μl phosphate buffer (0.01 mol/l pH 7.4) for 1 h and the buffer solution was centrifuged at 3,000 rpm for 20 min in at 4°C. IL-1β concentration in the eluted solution was analyzed by enzyme-linked immunosorbent assay (ELISA, Shanghai Haling Biological Technology Co., Ltd.).

### Alveolar Tissue Collection and Histology

After 8 weeks of traction, CBCT was taken again, and all Beagle dogs were sacrificed with an overdose of sodium pentobarbital. The maxillary bone with maxillary teeth was collected and fixed in the 10% formalin solution for 48 h. The hard tissue section and staining were processed at the Department of Oral Pathology of Beijing PLA General Hospital. Briefly, after 70, 80, 90, 95, and 100% alcohol dehydration (24 h for each liquid), the mixture of 3:1, 1:1, 1:3 alcohol and Technovit 7200 (volume ratio) was pre-impregnated (24 h for each liquid), and finally soaked in pure Technovit 7200 solution for 3 days, and then polymerized and embedded in embedding machine (Germany EXAKT-520). Tissue grinding slices were made by using a hard tissue-slicing machine (German EXAKT-300CP) to cut 200 microns thick slices. The slices were polished by grinding machine (German EXAKT-400CS). The final thickness of the grinding disc was determined by the thickness measurement induction device and micrometer. Each section was stained by MC NEAL's staining method (Liu et al., 2017). Each hard tissue slice contains the mesial root of the second premolar, and the section passed through the median section of the alveolar bone defect.

### Data Collection

#### Analysis of Newly Formed Bone

The alveolar bone defect area occupied by mineralized bone (lamellar and immature type), granules of bone filling material, and other tissues were determined by the use of a point-counting procedure and a lattice with 100 points (Zhang et al., 2011). The percentage of new bone as new bone formation ratio was calculated by dividing the area of bone tissue by the total area of the defect at each slice.

#### Residual Ratio of Bone Filling Material

The percentage of bone filling material was calculated by dividing the area of residual bone filling material by the total area of defect at each slice after traction of 2 months. We prepared the same size bone defect on a plastic model and filled the blood-soaked bone filling material to imitate the operation. Sections were made in the median section to estimate the percentage of bone defect area of these two kinds of filling materials (BioCaP or DBB) in the original condition. The residual rate of bone filling material was obtained by dividing the percentage of material in the section after traction by the percentage of original material (Liu et al., 2017).

#### Tooth Resorption Index Analysis

Image-Pro Plus 6.0 software was used to measure the area of resorption lacunae and the total root area on the mesial surface of the mesial root of the maxillary second premolar (orthodontic traction tooth) in each slice, and the root resorption index was calculated by using the following formula (Goldie and King, 1984).

$$\begin{array}{l} Root\;resorption\;index=\frac{Resorption\;area}{Total\;root\;area} \end{array}$$

#### Radiographic Gray Value of Bone Mineral Density

CBCT images were taken under anesthesia before surgery, 1 week after surgery, and 8 weeks after OTM. All CBCT images were imported to Mimics 15.0 software (Materialise Company, Belgium) and 3-dimension remodels built. We found the area of alveolar bone defect in the CBCT images 1 week after surgery and measured the volume data of the defect and filling materials. Through this localization, we also found the location of the alveolar bone defect in CBCT reconstruction images after 2 months of traction. We measured the Gray value, which serves as an indicator for bone density of the alveolar bone defects in all groups. By calculating the average radiographic gray value of bone defect area in 6 sections from buccal to palatal side, the parameters of bone mineral density were developed.

#### The Retraction Distance of Alveolar Ridge

The maxillary second premolars (orthodontic teeth) were found in the CBCT images before surgery, and the distances from the enamel-cementum boundary to the alveolar crest of the mesial side of the mesial root were measured in 3 sections from buccal to palatal side. The original height of alveolar crest was obtained by calculating the average of the above three values. And the height of alveolar crest after traction was calculated in the same way in the CBCT images 2 months after traction. The retraction distance of alveolar ridge was obtained by subtracting the height of alveolar ridge after traction from the original height of the alveolar ridge.

### Statistical Analysis

Data were submitted to SPSS ver. 19.0 (IBM Corporation, NY, U.S.A.) for analysis. All data are presented as mean values with the standard deviation (mean ± SD). A comparison of data within groups was performed using analysis of variance (ANOVA) followed by a post-Dunnett T3 test when ANOVA suggested a significant difference between groups. The significance level was set at *p* < 0.05.

## Results

### BioCaP Graft Enhanced Bone Regeneration and Alveolar Bone Defect Healing During OTM

**Figure 1:**
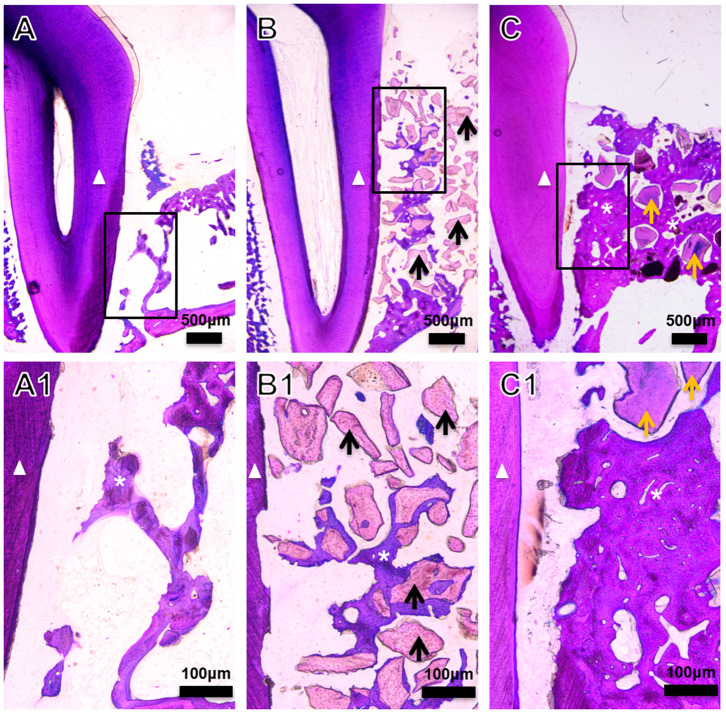
Representative histological images of the bone defect slices from **(A)** Control, **(B)** DBB, and **(C)** BioCaP group at week 8. **(A1–C1)** are high-resolution images of **(A–C)**, respectively. White triangle, dental root; asterisk, newly formed bone; black arrow, DBB; yellow arrow, BioCaP.

**Figure 2:**
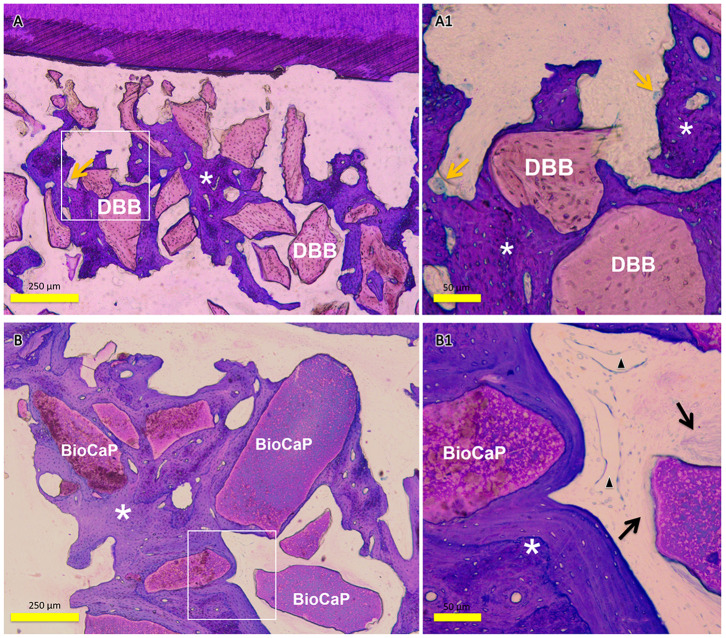
Representative histological images of the bone defect slice in DBB group and Bio-CaP group. **(A)** Bone remodeling process in DBB group at week 8. **(A1)** High-resolution image of **(A)**. **(B)** Bone remodeling process in BioCaP group at week 8. **(B1)** High-resolution image of **(B)**. Yellow arrows, multinucleated cells; asterisk, newly formed bone; black arrow, fibrous tissue; black triangle, blood vessel like structure.

The amount of encapsulated BMP2 in BioCaP granules and their release profile are well-documented in our previous studies (Liu et al., 2014, 2017, 2018; Zheng et al., 2014; Wang et al., 2019). Similarly, the extensive physicochemical characterization of BioCaP is available in our published papers (Liu et al., 2014, 2017; Wang et al., 2019). Histological images showed very less amount of newly formed bone in the control group (Figures 1A,A1). Bone regeneration was slightly improved in the DBB group (Figures 1B,B1). We observed robust bone regeneration in the BioCaP group compared to the control group and DBB group (Figure 1). High-resolution images illustrated newly formed bone nearby or around the BioCaP granules. Dental root erosion was observed in the DBB group (Figure 1). The area of newly formed bone tissue was evidently less in DBB-group compared to the BioCaP group. Multinucleated osteoclast-like cells were observed only in the DBB group, which explains the dental root erosion in the DBB group (Figures 2A,A1). The absence of multinucleated osteoclast-like cells in the BioCaP group indicates the less immunogenicity and higher biocompatibility of BioCaP granules. A densely stained newly formed bone was observed in the BioCaP group (Figures 2B,B1). Similarly, blood vessels like structures were observed only in BioCaP group (Figures 2B,B1). In the control group, most of the bone defect area was filled with fibrous connective tissue (data not shown).

**Figure 3:**
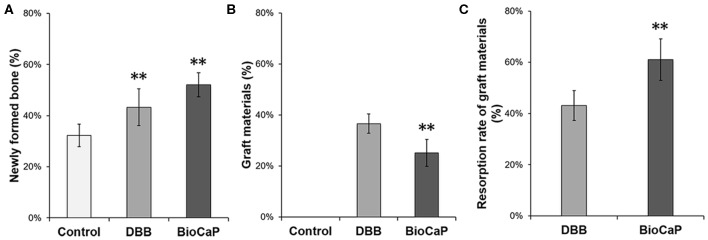
BioCaP grafting enhanced bone formation and degradation of graft material. **(A)** Percentage of newly formed bone at week 8. **(B)** Percentage of remaining graft materials at week 8. **(C)** The resorption rate of graft materials at week 8. Data are presented as mean ± SD, from six independent experiments. Significant effect of the treatment, **p < 0.01.

Quantitative analysis data showed 1.30-fold higher newly formed bone percentage in the DBB group compared to the control group (Figure 3A). In the BioCaP group, newly formed bone percentage was increased by 1.61-, and 1.25-fold compared to the control group and DBB group, respectively. Remaining graft material at week 8 was 1.45-fold higher in the DBB group compared to the BioCaP group (Figure 3B). Resorption rate of graft material was 1.42-fold higher in the BioCaP group compared to the DBB group (Figure 3C).

### BioCaP Graft Enhanced Bone Mineral Density in the Alveolar Defects

**Figure 4:**
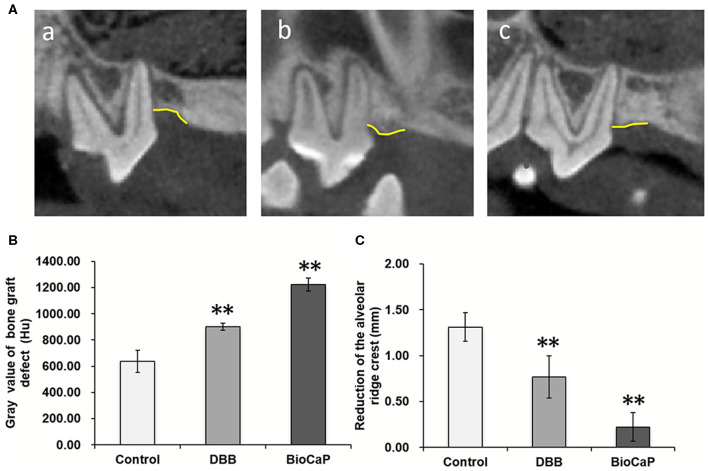
**(A)** Representative CBCT images at week 8, (a) Control, (b) DBB, and (c) Bio-CaP group. **(B)** Gray value of bone graft defect (Hu). **(C)** Reduction of the alveolar ridge crest (mm). Data are presented as mean ± SD, from six independent experiments. Significant effect of the treatment, **p < 0.01. Yellow line, alveolar bone ridge.

CBCT images were taken at baseline, 1 week after grafting, and 8 weeks after OTM (Supplementary Figure 1). We measured the Gray value, which serves as an indicator for the bone density of the alveolar bone defects in all groups. The bone density in the BioCaP group was 1.92-, and 1.36-fold higher compared to the control and DBB group, respectively. Both histological and CBCT images showed distinct bony alveolar ridge in the BioCaP group compared to the DBB group (Figures 1, 4). In contrast, almost empty alveolar defects in the control group and mostly DBB filled alveolar defect were observed in the DBB group (Figures 1, 4).

### BioCaP Graft Alleviated Root Resorption and Alveolar Retraction

**Figure 5:**
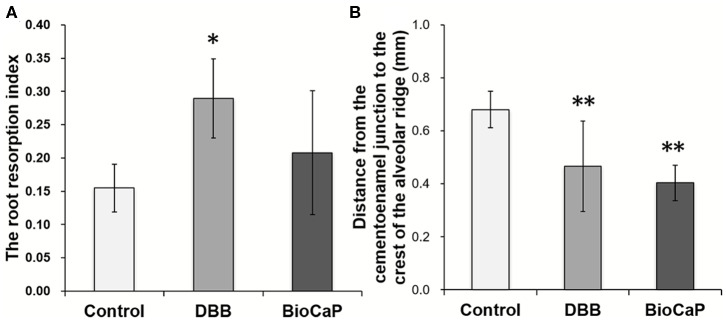
Bio-CaP didn't affect the dental root resorption and maintained the distance from the enamel cementum to the crest of the alveolar ridge. **(A)** The root resorption index at week 8. **(B)** The distance from the enamel cementum to the crest of the alveolar ridge at week 8. Data are presented as mean ± SD, from six independent experiments. Significant effect of the treatment, *p < 0.05, and **p < 0.01.

The root resorption index in the DBB group was 1.87-fold higher compared to the control group (Figure 5A). Although the higher trend of the root resorption index was observed in the DBB group compared to the BioCaP group, the effect was not statistically significant. The distance between the enamel cementum and the crest of the alveolar ridge in the control group was 1.45-, and 1.69-fold higher compared to DBB and BioCaP group, respectively (Figure 5B). There was no significant difference in distance from the enamel cementum to the crest of the alveolar ridge between the DBB and the BioCaP group (Figure 5B). BioCaP did not affect root resorption and resulted in the minimum distance between the enamel cementum and the crest of the alveolar ridge compared to the control and DBB group.

### BioCaP Graft Favored Maintaining the Periodontal Attachment

Periodontal probing depth of orthodontic teeth was measured every 2 weeks during orthodontic traction. The periodontal probing depth tends to increase in the control group compared to the DBB group, but the difference was not statistically significant. The PDD was lowest in the BioCaP group compared to the control group and DBB group. However, the periodontal probing depth in the control group was significantly greater than the BioCaP group only at week 8. There was no significant difference in periodontal probing depth between DBB and BioCaP group at all the tested time points (Table 1).

**Table 1:** Periodontal probing depth (mm) of orthodontic teeth.

**Group**	**Control**	**DBB**	**BioCaP**
0 week	1.67 ± 0.52	1.58 ± 0.39	1.58 ± 0.38
2 weeks	2.25 ± 0.52	1.92 ± 0.20	1.50 ± 0.55
4 weeks	2.08 ± 0.66	1.75 ± 0.76	1.42 ± 0.49
6 weeks	1.92 ± 0.58	1.42 ± 0.38	1.33 ± 0.61
8 weeks	2.17 ± 0.75*	1.67 ± 0.41	1.25 ± 0.42

Data are presented as mean ± SD from six independent experiments (n = 6). Significant difference compared to BioCaP group,

* *p < 0.05*.

### The Differences in OTM Value Were Not Significant Among the Tested Groups

**Figure 6:**
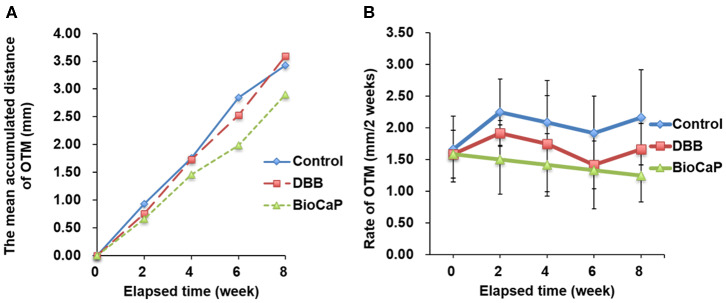
The distances of OTM were less in Bio-CaP group compared to other groups. **(A)** The mean accumulated distance of OTM at 8 weeks, **(B)** Rate of OTM at 2 weeks interval. Data are from six independent experiments.

OTM was measured every 2 weeks for all groups during Orthodontic traction. All sites of alveolar bone defect and extraction healed smoothly after the surgical procedure. There was no incidence of infection, suppuration, or death. After the activation of the orthodontic device, the orthodontic teeth (bilateral maxillary second premolars) started to move in the mesial direction. CBCT images before and after orthodontic exertion revealed that the mesial root of all maxillary second premolars was located within the area of alveolar bone defects (Figure 4A). The total OTM was 2.90 ± 0.84 mm in BioCaP group, 3.59 ± 1.25 mm in the DBB group, and 3.42 ± 1.55 mm in the control group (Table 2). There was no significant difference in the distance traveled during OTM among the three groups (Figures 6A,B).

**Table 2:** OTM at 2 weeks interval.

**Group**	**1–2 weeks**	**3–4 weeks**	**5–6 weeks**	**7–8 weeks**	**Total**
Control	0.93 ± 0.33	0.83 ± 0.41	1.09 ± 0.58	0.58 ± 0.26	3.42 ± 1.55
DBB	0.75 ± 0.29	0.98 ± 0.30	0.80 ± 0.37	1.06 ± 0.59	3.59 ± 1.25
BioCaP	0.65 ± 0.19	0.80 ± 0.29	0.53 ± 0.26	0.92 ± 0.23	2.90 ± 0.84

*Data are presented as mean ± SD from six independent experiments (n = 6)*.

### BioCaP Graft Alleviated IL-1β Concentration in GCF

**Figure 7:**
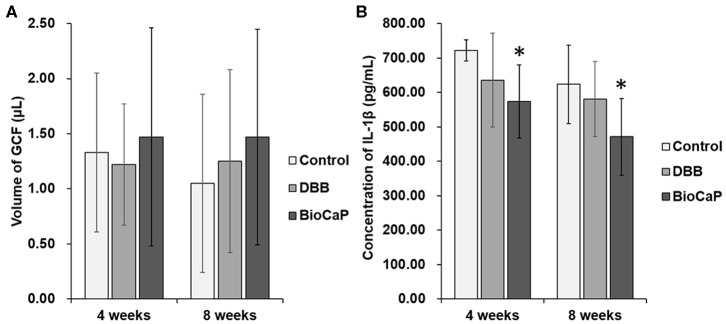
In BioCaP group the volume of GCF was not affected but and IL-1β concentration was reduced. **(A)** Volume of GCF and **(B)** Concentration of IL-1β in GCF at week 4 and 8 of OTM. Data are presented as mean ± SD, from six independent experiments. Significant effect of the treatment, *p < 0.05.

DBB or BioCaP graft did not affect GCF volume in the OTM site at week 4 and 8 (Figure 7A). However, the concentration of IL-1β in GCF was the lowest in the BioCaP group compared to the control and DBB group (Figure 7B). DBB group failed to reduce the IL-1β concentration in GCF compared to the control group. Our results indicate the anti-inflammatory or less immunogenic property of the BioCaP scaffolds.

## Discussion

**Figure 8:**
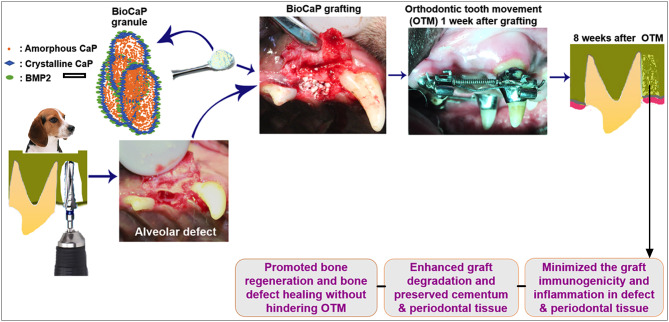
Scheme illustrating the procedure and main findings of this study.

The impact of various xenograft or biomaterial grafts in the orthodontic treatment had been reported (Hossain et al., 1989, 1996; Araujo et al., 2001, 2009; Reichert et al., 2010). Xenograft or biomaterial grafts facilitates orthodontic treatment but has inferior bone regenerative potential compared to auto-bone graft. This might be mainly due to the lack of osteoinductive potential of the bone grafts. DBB materials, such as Bio-Oss^R^, are acellular products from bovine bone, which has been widely used to augment alveolar ridge. However, the osteoinductivity and biodegradability of DBB are unsatisfactory (Araujo et al., 2001; Ahn et al., 2014). In this study, we aimed at evaluating whether the biomimetic and osteoinductive BioCaP could be better graft material for alveolar bone defect in sequent OTM compared to the commonly used DBB xenograft. BioCaP graft robustly enhanced bone regeneration and graft biodegradation compared to DBB. Moreover, the BioCaP graft did not influence the OTM rate and was less immunogenic compared to DBB, but preserved the orthodontic tooth root erosion, reduced inflammation, and maintained the periodontal attachment as illustrated in Figure 8. The results of our study indicate BioCaP as a promising graft material for alveolar or periodontal bone regeneration during orthodontic treatment.

Fabrication technique of BMP2-functionalized BioCaP granules, physicochemical properties, *in vitro* and *in vivo* BMP2 release profile, biocompatibility, osteoconductivity, osteoinductivity, and bone defect healing potential of BioCaP had been extensively investigated in our previous studies (Hunziker et al., 2012; Liu et al., 2014, 2017, 2018; Zheng et al., 2014; Wang et al., 2017, 2019). DBB xenograft lacks osteoinductivity but has been commonly used as alveolar defect filling material during OTM treatment. In this study, BioCaP robustly enhanced alveolar bone defect healing during OTM. Histological images and CBCT images clearly indicate the higher amount of newly formed bone and distinct alveolar ridge in the BioCaP group compared to the DBB group. Alveolar bone defect healing process and duration in the dog are similar to that in humans (Giannobile et al., 1994). Therefore, we used orthodontic traction in the surgical alveolar bone defect in Beagle dogs. Immediate orthodontic traction after alveolar bone grafting accelerates OTM and bone mineralization in the tension side (Ahn et al., 2014). Furthermore, Seifi and Ghoraishian (2012) concluded that tooth movement could start immediately before healing of the grafting site. In this study, we started orthodontic traction 1 week after BioCaP or DBB grafting, which coincides with the early stage of woven bone formation (Ahn et al., 2014). Orthodontic treatment facilitates the healing of not only woven bone but also alveolar bone (Araujo et al., 2001; Reichert et al., 2010). In this study, only BioCaP grafting showed effective bone regeneration and alveolar bone defect healing in comparison to empty defect or DBB grafting (Figures 1–4). Our result indicates the BioCaP as a cost-effective graft material to fill alveolar bone defects during orthodontic treatment.

OTM is affected by the cellular and mineral components of the periodontium, which are influenced by the grafting material and OTM timing. Higher OTM is observed during the immediate application of orthodontic force after implantation of graft compared to the application of orthodontic force after 2 or 12 weeks after grafting (Ahn et al., 2014). Orthodontic traction started 2 weeks after the implantation of bone graft in alveolar bone defect has no detrimental effects on orthodontic movement (Cardaropoli et al., 2006). Early traction force allows orthodontic teeth movement into the bone graft area. In this study, similar OTM distance and rate of OTM were observed in control, DBB, and BioCaP group, indicating that both DBB and BioCaP did not obstruct the OTM during 8 weeks. But Machibya et al. (2018) reported higher OTM in the DBB group compared to CaP or no graft group. This discrepancy might be caused by the different starting times of orthodontic traction, i.e., at least 1 month after grafting and OTM timing of 6 weeks (Machibya et al., 2018). Our results indicate that early orthodontic traction (1 week after grafting) in BioCaP grafted defect enhanced alveolar bone defect healing without hampering OTM.

Biomaterial immunogenicity directly affects bone regeneration via inducing foreign body reaction-mediated inflammation in the defect site (Velard et al., 2013; Klopfleisch and Jung, 2017). The proinflammatory environment in the defect will recruit more immune cells such as monocytes and macrophages inducing the formation of multinucleated giant cells and osteoclasts (Velard et al., 2013). Multinucleated cells not only resorb the native bone or tooth cementum but also inhibit bone regeneration via further upregulation of pro-inflammatory cytokines. In this study, multinucleated osteoclast-like cells were observed only in the DBB group but not in the control and BioCaP group. Dental root resorption is another important issue during biomaterial grafting and/or orthodontic treatment. BMP2-loaded graft had shown orthodontic tooth root resorption in the dose (BMP2) dependent manner (Kawamoto et al., 2002, 2003). Dose of 40 μg BMP2/100 μl graft resorbs the dentine, and 10 μg BMP2/100 μl graft only resorbs the cementum of orthodontic tooth root (Kawamoto et al., 2002, 2003), which might be the effect of burst release of BMP2 from the graft materials. In this study, the root surface was not in direct contact with the graft material during grafting, but it might come in contact with graft material during OTM. We found that 14 μg BMP2 in 100 μl of BioCaP did not resorb the cementum of orthodontic tooth root. This might be the effect of sustained controlled release of a low dose of BMP2 from BioCaP around the tooth root. Another possible reason for cementum erosion of orthodontic tooth root in the DBB group could be the higher activity of the DBB immunogenicity-induced multinucleated osteoclast-like cells. Similarly, our previous study reported the higher numbers of multinucleated osteoclast-like cells in DBB-grafted femoral defect compared to in BioCaP grafted defect (Wu et al., 2011; Liu et al., 2017). Our study revealed that the BioCaP graft protects the erosion of orthodontic tooth root.

Angiogenesis facilitates bone and periodontal tissue regeneration via osteogenesis-angiogenesis coupling (Huang et al., 2015; Hu and Olsen, 2016). In this study, histological images showed higher numbers of blood vessel-like structures in the BioCaP group. Similarly, bone regeneration and alveolar defect healing were enhanced in the BioCaP group. This indicates the possibility of the BioCaP-mediated osteogenesis-angiogenesis coupling. Degradation of graft materials in a certain duration is essential for proper bone healing. In our previous study, BioCaP showed excellent degradation properties during bone defect healing (Liu et al., 2017). In this study, both the graft degradation rate and bone regeneration were higher in the BioCaP group. Spontaneous dissolution in body fluid and higher cellular activity-based resorption controls the degradation of BioCaP graft (Hunziker et al., 2012; Zheng et al., 2014). The higher resorption of graft material enhances, and the higher amount of newly formed bone inhibits OTM. In this study, the OTM rate was slightly less but statistically not significant in the BioCaP group compared to other groups. This result might be the combined effect of higher bone formation and higher graft degradation in the BioCaP group.

The higher periodontal probing depth indicates a higher degree of periodontal damage (Preshaw, 2015). In this study, only the BioCaP but not DBB graft was able to reduce periodontal probing depth compared to the control group. This indicates the ability of BioCaP to protect the periodontal damage during OTM. The higher level of proinflammatory cytokines in alveolar defect inhibits bone defect healing. Similarly, the higher level of proinflammatory cytokines in GCF causes inflammation and destroys the periodontal tissue. IL-1β is a key proinflammatory cytokine that causes periodontal damage (Cheng et al., 2020). In this study, only the BioCaP but not the DBB graft significantly reduced the level of IL-1β in GCF compared to the control at week 4 and 8. The less immunogenicity of BioCaP graft may reduce the recruitment of immune cells not only in the alveolar defect but also around the periodontal tissue of orthodontic tooth alleviating the concentration of inflammatory cytokines in GCF.

In this study, we analyzed the efficacy of biomimetic and osteoinductive BioCaP graft on alveolar bone defect healing during orthodontic traction. We used the standard protocol to fabricate the BioCaP and adopted the established dog model for alveolar bone defect and orthodontic traction. This experiment model highly resembles the human alveolar bone defect healing during orthodontic treatment. The autologous bone graft is the gold standard for alveolar bone defect healing. A limitation of this study is that we did not compare the effect of BioCaP with the autologous bone graft. Hybrid polymer based biomaterials such as a combination of biodegradable polymer, osteoconductive CaP/HAP, and osteoinductive bioglass/magnesium could be effective for alveolar bone regeneration during OTM (Lei et al., 2019). Moreover, periodontal tissue and periodontal ligaments should be monitored during orthodontic treatment and/or alveolar bone defect healing. Another limitation of this study is that we evaluated bone formation and OTM only up to 8 weeks. To address these issues, future studies that compare the effect of BioCaP with auto bone graft on alveolar bone defect healing during orthodontic traction in 12 and 16 weeks are recommended.

## Conclusion

In conclusion, the results of this study showed that the biocompatible, osteoconductive, osteoinductive BioCaP graft is more effective on alveolar bone defect healing and protection of orthodontic tooth root during orthodontic treatment compared to commonly used DBB. Biomimetic BioCaP granules carrying a low dose of BMP2 and giving slow and sustained release of BMP2 show less immunogenicity, high biodegradability, and proangiogenic potential. BioCaP graft reduces the inflammation in GCF and periodontal probing depth in the orthodontic tooth. Our results suggest BioCaP as a cost-effective graft material for filling the bone defect during orthodontic treatment.

## Data Availability Statement

The datasets generated for this study are available on request to the corresponding author.

## Ethics Statement

The animal study was reviewed and approved by the Ethics committee of the Zhejiang University Laboratory Animal Center.

## Author Contributions

JS, GW, JP, and SJ: study concept and design. SJ and TL: data acquisition and analysis. SJ and WL: performed experiments. SJ and XF: animal experiments. SJ, JS, and TL: manuscript preparation. JP and WL: manuscript review. GW: preparation of BioCaP granules. All authors: read and approved the submitted version.

## Conflict of Interest

The authors declare that the research was conducted in the absence of any commercial or financial relationships that could be construed as a potential conflict of interest.

## Supplementary Material

Supplementary Figure 1 Representative CBCT images taken at baseline, 1 week after grafting, and 8 weeks after OTM in control, DBB, and BioCaP group.
