# Supplementary figures and images for: BMP2-Functionalized Biomimetic Calcium Phosphate Graft Promotes Alveolar Defect Healing During Orthodontic Tooth Movement in Beagle Dogs
Source: Front Bioeng Biotechnol. 2020 May 29;8:517. doi: 10.3389/fbioe.2020.00517 (PMC7272671; doi:10.3389/fbioe.2020.00517)

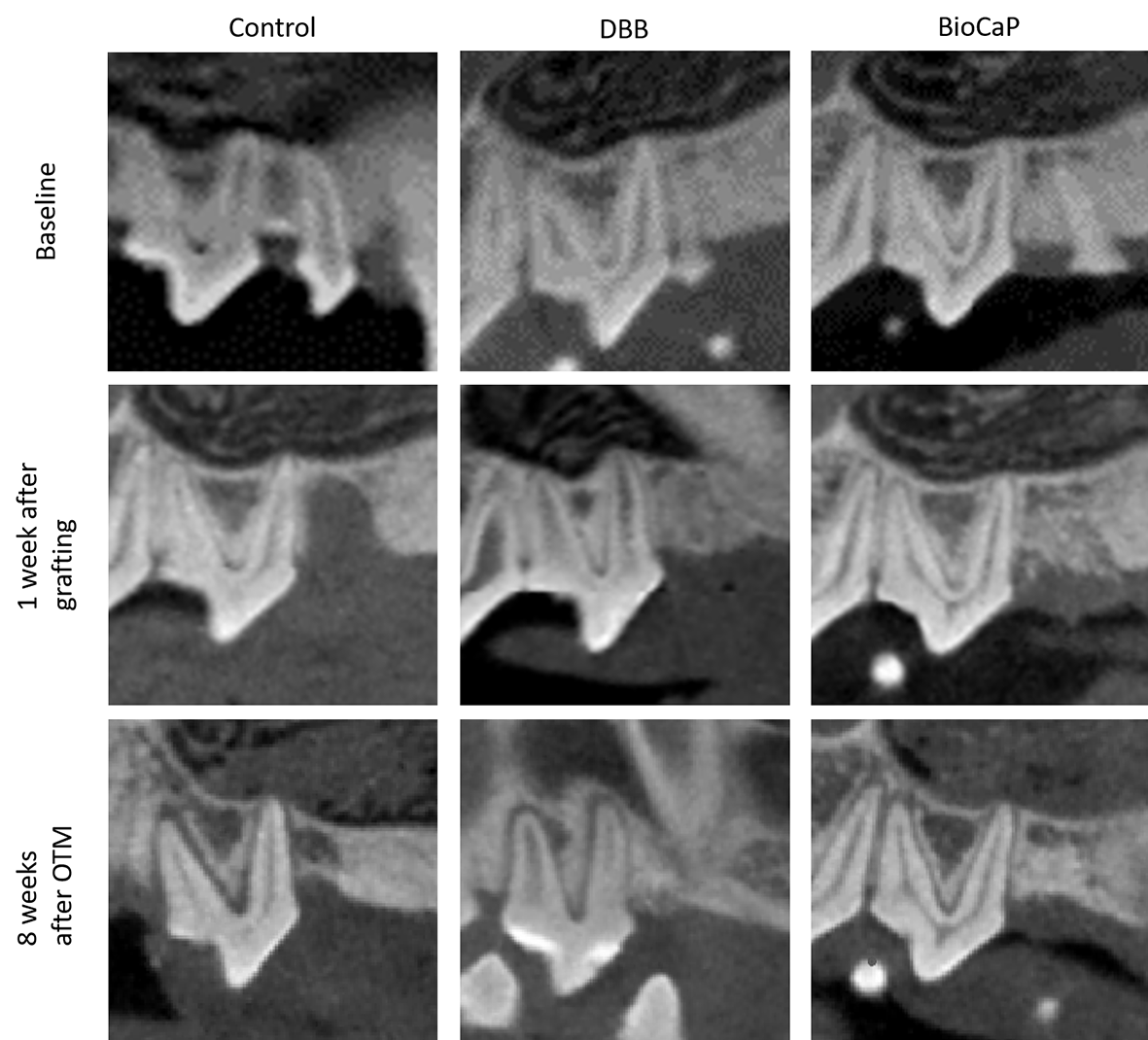

Supplement: Supplementary Figure 1 — Representative CBCT images taken at baseline, 1 week after grafting, and 8 weeks after OTM in control, DBB, and BioCaP group. [file Image_1.tif]
